# Errata in January and February Nos

**Published:** 1868

**Authors:** 


### ERRATA IN JANUARY AND FEBRUARY NOS.

P. 471, l. 7 from top, after "specific" read "name;" l. 8, before "stems" read "scabrous;" p. 497, l. 17 from top, instead of "chicken" read "checker;" l. 23, after "usually" read "snowy white, sometimes tinged with red, densely hairy within, two on each ovary;" l. 24, read "insipid;" p. 498, l. 7 from bottom, instead of "best, best," read "last, best;" p. 449, l. 7 from top, instead of "prepared" read "preferred;" p. 500, l. 16 from top, instead of "Gelsemi*n*um" read "Gelsemium;" p. 502, l. 2 from bottom, instead of "9600" read "1600;" p. 469, l. 26, for "oz." read "oj.;" p. 517, l. 16, instead of "for no higher motive" read "from," etc.; p. 518, l. 7, fer "physical" read "psychical;" l. 9 and 10, for "affirmation" read "affirmative;" l. 14, for "providence" read "revelation;" l. 23, for "prevents" read "perverts."

